# MASP-1 Induced Clotting – The First Model of Prothrombin Activation by MASP-1

**DOI:** 10.1371/journal.pone.0144633

**Published:** 2015-12-08

**Authors:** Lorenz Jenny, József Dobó, Péter Gál, Verena Schroeder

**Affiliations:** 1 University Clinic of Haematology, Haemostasis Research Laboratory, University Hospital Bern, Bern, Switzerland; 2 Department of Clinical Research, University of Bern, Bern, Switzerland; 3 Institute of Enzymology, Research Centre for Natural Sciences, Hungarian Academy of Sciences, Budapest, Hungary; University Medical Center Utrecht, NETHERLANDS

## Abstract

Mannan-binding lectin-associated serine protease-1 (MASP-1), a protein of the complement lectin pathway, resembles thrombin in terms of structural features and substrate specificity. Due to its interplay with several coagulation factors, it has the ability to induce fibrin clot formation independent of the usual coagulation activation pathways. We have recently shown that MASP-1 activates prothrombin and identified arginine (R) 155, R271, and R393 as potential cleavage sites. FXa cleaves R320 instead of R393, and thrombin cleaves R155 and R284 in prothrombin. Here we have used three arginine-to-glutamine mutants of prothrombin, R271Q, R320Q, R393Q and the serine-to-alanine active site mutant S525A to investigate in detail the mechanism of MASP-1 mediated prothrombin activation. Prothrombin wildtype and mutants were digested with MASP-1 and the cleavage products were analysed by SDS-PAGE and N-terminal sequencing. A functional clotting assay was performed by thrombelastography. We have found that MASP-1 activates prothrombin via two simultaneous pathways, either cleaving at R271 or R393 first. Both pathways result in the formation of several active alternative thrombin species. Functional studies confirmed that both R393 and R320 are required for prothrombin activation by MASP-1, whereas R155 is not considered to be an important cleavage site in this process. In conclusion, we have described for the first time a detailed model of prothrombin activation by MASP-1.

## Introduction

The complement system is a central part of the innate immune system and has a crucial function in the clearance of pathogens from the circulation. The lectin pathway is one of three possible ways of the complement system to encounter threats due to infection. It recognises its targets by binding of mannan-binding lectin (MBL) or ficolins to specific patterns on foreign and/or altered surfaces [1]. This leads to the activation of the MBL-associated serine proteases (MASPs) MASP-1, MASP-2, and MASP-3 which then produce C3 convertase via C2 and C4 cleavage (reviewed in [2]). MASP-1 is now considered the central enzyme in the early lectin pathway as it is able to autoactivate, then activates both MASP-2 and MASP-3 [3].

Beside its central role in the lectin pathway, MASP-1 has been shown to interact with the coagulation system [4]. The serine protease domain of MASP-1 is more closely related to thrombin and trypsin than to the rest of the C1r/C1s/MASP family of serine proteases [5]. Its wide substrate binding cleft and the consequential broad substrate specificity allows it to interact with several proteins of the coagulation system including coagulation factor XIII (FXIII), fibrinogen and prothrombin [6–8]. Furthermore MASP-1 is inhibited in the presence of glycosaminoglycans more efficiently by antithrombin than by C1-inhibitor [5,9].

*In vivo* evidence for a role of MASP-1 in coagulation came from MASP-1 and MBL knockout mice. Upon tail tip excision the knockout mice showed prolonged bleeding time [10] and a significant decrease in FeCl_3_-induced thrombogenesis [11].

We have shown recently that MASP-1 is able to trigger clot formation in whole blood and platelet poor plasma, and that the effects of MASP-1 and thrombin are additive, although the effect of MASP-1 is weaker and lags behind the effect of thrombin, and that MASP-1 induced clotting depends on the presence of prothrombin [7,8]. Thus, we proposed that MASP-1 induces clotting mainly via prothrombin activation and identified three potential cleavage sites: arginine 155 (R155), R271 and R393 [8].

**Fig 1:**
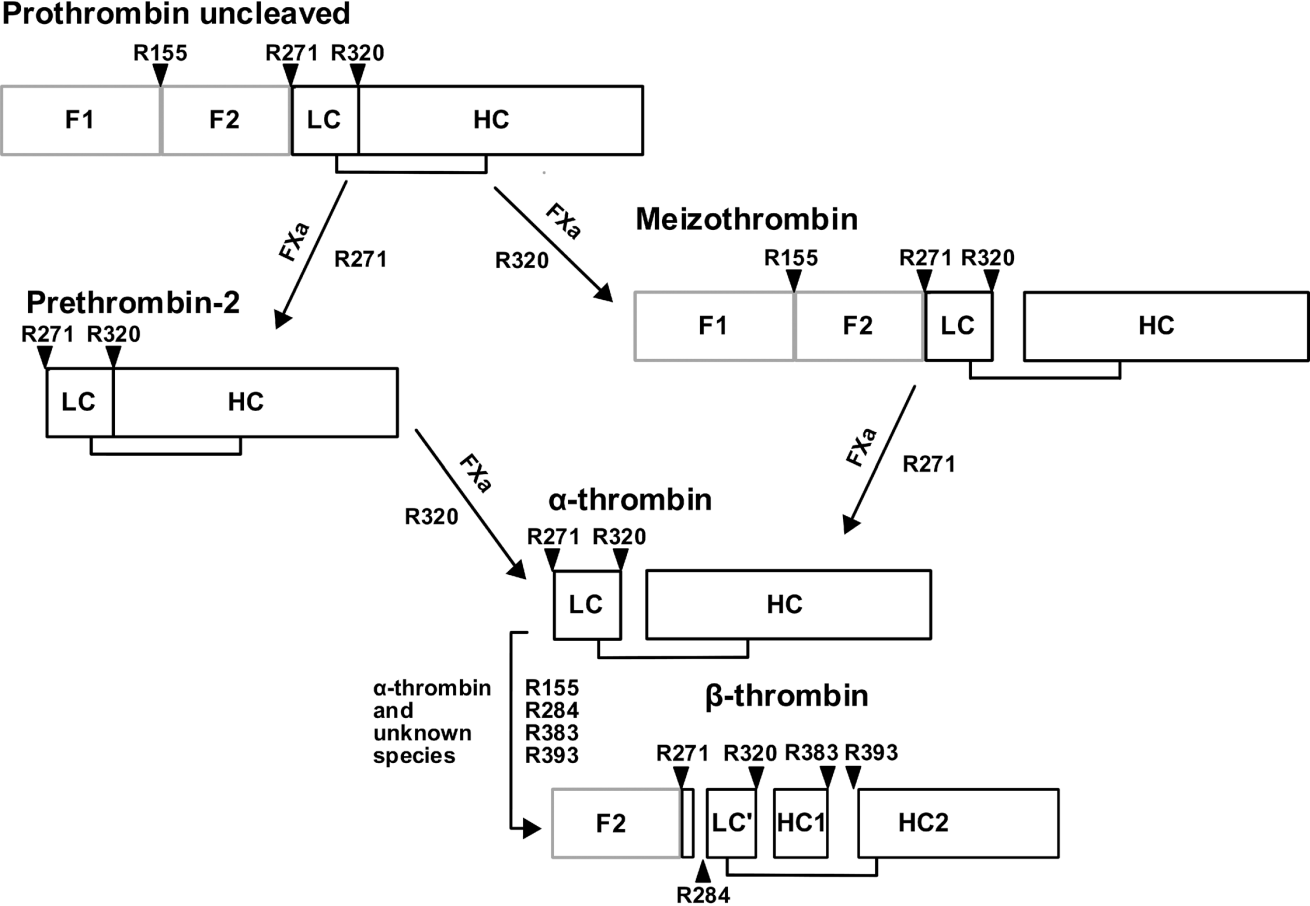
FXa mediated prothrombin cleavage. Prothrombin activation by FXa. FXa cleaves prothrombin either at R271 or at R320. Intermediates gain their activity by cleavage at R320 and are then able to perform further cleavages at R155, R284, R383 and R393.

In the course of FXa mediated prothrombin activation (Fig 1), various thrombin species occur. Alpha-thrombin is the central enzyme in the coagulation cascade, and it is produced by cleavage at R271 and R320, whereby the site to be cleaved first depends on the absence/presence of the prothrombinase complex [12]. Meizothrombin (mIIa) is the first intermediate to be produced in presence of the prothrombinase complex and arises by cleavage of prothrombin at R320. In absence of the prothrombinase complex and in fluid phase, prothrombin is cleaved first at R271 to release prothrombin fragment F1.2 and prethrombin-2. Further (autolytic) degradation of α-thrombin by cleavage at R284, R383, and R393 leads to the formation of β-thrombin which has significantly reduced clotting activity [13].

MASP-2 was reported to cleave prothrombin in a similar fashion as FXa [14], whereas thrombin cleaves prothrombin at different sites (R155 and R284) than FXa [13,15]. In MASP-1 mediated cleavage of fibrinogen and FXIII, however, MASP-1 does not cleave these substrates in the exact same way and/or efficiency as thrombin does [6].

It is interesting that MASP-1 mediated prothrombin activation seems to involve cleavage sites of both FXa and thrombin mediated proteolytic processes. This would imply that a new model, containing at least one new thrombin species, is necessary to explain clotting induced by MASP-1. Therefore, the aim of our present study was to elucidate the mechanism and the order of MASP-1 mediated prothrombin cleavage. We generated and expressed a novel prothrombin mutant (R393Q) which we used alongside with the prothrombin mutants R271Q and R320Q [12] in digestion and functional experiments.

## Materials and Methods

### 2.1. MASP-1 protein

We used a recombinant MASP-1 catalytic fragment (rMASP-1cf) in this study, since it is not yet possible to produce or purify sufficient amounts of pure and stable full-length MASP-1. This truncated form of MASP-1 consists of the CCP1-CCP2-SP domains while it lacks the N-terminal CUB1-EGF-CUB2 domains [16]. We have recently shown that rMASP-1cf and full-length MASP-1 show the same effects on clot formation [8].

### 2.2. Prothrombin mutants

In the prothrombin mutants, the respective arginine (R) was replaced by glutamine (Q) in order to abolish individual cleavage sites. The prothrombin mutants R271Q, R320Q and S525A were a kind gift from Prof. Sriram Krishnaswamy (University of Pennsylvania, Philadelphia, USA). R271Q and R320Q are mutants of the FXa cleavage sites. S525A is a mutant of the active site of thrombin (it corresponds to S195A according to chymotrypsin numbering). To generate the prothrombin mutant R393Q a pcDNA 3.1 (+) plasmid holding the sequence for wildtype prothrombin [17], also a kind gift from Prof. Krishnaswamy, served as a template. The Arg393Gln mutation was introduced with mutagenic primers and the QuickChange mutagenesis kit (Stratagene, La Jolla, USA). Sequencing assured that the mutated prothrombin construct was correct.

HEK 293 cells (CRL-1573, directly obtained from ATCC, Manassas, USA) were cultured in D-MEM+/+ (containing 10% FCS, 1% Hepes, 1% non-essential amino acids, 1% sodium pyruvate; Life Technologies, Carlsbad, USA). Transfection was performed with 7.5 μg of plasmid DNA and 10 μl of Lipofectamine 3000 per 5*10^5^ cells for 24h in Opti-MEM (both Life Technologies). Cells were then transferred into D-MEM+/+ and selected by geneticin (G418, 0.5mg/ml, Life Technologies). The amount of produced R393Q prothrombin by stable cell lines was assessed by ELISA (Prothrombin (Factor II) human Elisa kit; Abcam, Cambridge, UK). Selected cell lines were expanded in T150 flasks (Opti-MEM, supplemented with 10 μg/ml reduced vitamin K; Sigma-Aldrich, St. Louis, USA) and the conditioned medium was harvested daily for 5 days and immediately treated with 5 mM benzamidine and stored at -20°C.

For the purification, the conditioned medium was thawed in a water bath and centrifuged for 5 min at 1600 rpm. The supernatant was filtered through a 0.2 μm filter (Thermo Scientific, Waltham, USA) before applied to a Q-sepharose anion exchange column (HiTrap Q FF, GE Healthcare, Chalfont St Giles, UK) equilibrated with 20 mM Hepes and 1 mM benzamidine, pH 7.5. After washing with 20 mM Hepes, pH 7.5, R393Q prothrombin was eluted with 20 mM Hepes (pH 7.5) and a gradient of 0-1M NaCl, at a flow rate of 5 ml/min. Protein fractions were pooled and applied to a high-resolution Q sepharose anion exchange column (HiTrap Q HP, GE Healthcare) equilibrated with 20mM Hepes, 0.5 M NaCl and 1 mM benzamidine, pH 7.5. After washing with 20 mM Hepes, pH 7.5, the protein was eluted with 20 mM Hepes, 1 M NaCl, pH 7.5, and prothrombin containing fractions were pooled and stored at -20°C. The benzamidine did eluted before the prothrombin fraction and was therefore not present in the purified prothrombin samples. Both ion exchange chromatography steps were performed on an ÄKTA purifier 10 FPLC (fast protein liquid chromatography) device (GE Healthcare).

### 2.3. Thrombelastography experiments

Thrombelastographic measurements were performed on a rotation thrombelastometry system (ROTEM^®^, Tem International, Munich, Germany). Wildtype or mutant prothrombin (final concentration of 100 μg/ml (1.4 μmol/l)) was added to fibrinogen (2 mg/ml (5.8 μmol/l), Hyphen BioMed) in TBS. Upon addition of rMASP-1cf (80 μg/ml (1.76 μmol/l)) or FXa as a control (5 μg/ml (114 nmol/l)), respectively, measurements were started and clotting times (CT), representing the lag time until the onset of clot formation, were recorded during 1 hour.

### 2.4. Prothrombin cleavage studies

Cleavage studies were performed as described earlier [8]. Briefly, prothrombin wildtype (Hyphen BioMed, Neuville-sur-Oise, France), or R271Q, R320Q, R393Q or S525A mutants (1.4 μmol/l corresponding to 100 μg/ml) were incubated at 37°C for up to 90 min (up to 120 min to assess the cleavage rate of full-length prothrombin) with rMASP-1cf (1.76 μmol/l corresponding to 80 μg/ml) in a final volume of 50 μl Tris-buffered saline (TBS, 50 mmol/l Tris, 100 mmol/l NaCl, pH 7.4). After incubation, cleavage products were separated by SDS-PAGE and the gels were stained with Coomassie. For N-terminal sequencing, bands were transferred onto a polyvinylidene difluoride (PVDF) membrane by Western blotting, excised from the membrane and underwent five cycles of Edman degradation and N-terminal sequencing.

### 2.5. Kinetic analysis

Wildtype and S525A mutant prothrombin cleavage reactions were prepared as described in paragraph 2.4. The gels were scanned with an Odyssey imaginer (Li-Cor, Lincoln, USA) and the densitometric scans were quantified using Prism software (GraphPad Software, La Jolla, USA). The data was fitted by nonlinear regression using the equation Y = Y0+A*exp(-K*X), where X is the time and Y are the corresponding densitometric values. The calculated observed first order rate constant (k observed) is expressed by k_obs_/[E] where [E] stands for the total enzyme concentration.

## Results

Recently, we have shown that MASP-1 is able to induce clot formation by an alternative prothrombin activation and identified R155, R271, and R393 as the putative cleavage sites [8]. In the present study we have used prothrombin mutants in which individual cleavage sites are abolished, and we are now able to elucidate the pathways of prothrombin activation by MASP-1 leading to active thrombin species.

### 3.1. Prothrombin mutants

Beside the three prothrombin mutants R271Q, R320Q [17] and S525A, we also used R393Q which we expressed in our laboratory. We assessed that our transfected Hek 293 cell lines expressed prothrombin in the range of 4.9–5.8 μg/ml per 24h at confluency.

In order to test the functionality of the three prothrombin mutants in terms of their clot formation ability, they were incubated with fibrinogen (2 mg/ml) and FXa (5 μg/ml), then measured on a thrombelastograph (Table 1). When prothrombin R271Q was activated with FXa it showed clotting times similar to wildtype prothrombin, whereas the mutant forms R393Q and R320Q showed much longer clotting times. These results suggest that the cleavage site R320 is crucial for FXa mediated prothrombin activation, while R393 plays a minor role in this process. The functionality of all mutant prothrombin forms could be confirmed as their activated forms led to clot formation in all three cases.

**Table 1:** Clotting times of prothrombin forms, incubated with fibrinogen and either FXa or MASP-1.

Prothrombin	FXa	MASP-1
Wildtype	15 min ±0.9	36 min ±1.3
R271Q	13 min ±1.7	51 min ±2.9
R320Q	52 min ±2.1	-
R393Q	25 min ±2.1	-
S525A	-	-

Protein concentrations used are: prothrombin forms 100 μg/ml (1.4 μmol/l), FXa 5 μg/ml (114 nmol/l) and MASP-1 80 μg/ml (1.76 μmol/l). The clotting time is shown as mean value ± standard deviation (n = 3).

### 3.2. Functional studies with MASP-1

In order to test if any of the prothrombin mutants would induce fibrin formation when activated with MASP-1, we incubated the different prothrombin mutants with fibrinogen and activated with MASP-1 (80 μg/ml) (Table 1). Incubation of prothrombin mutant R271Q with MASP-1 led to clot formation, however, the onset was much later than with wildtype prothrombin. MASP-1 failed to induce clot formation by activation of the R393Q mutant. This implicates that R393 is a crucial cleavage site for MASP-1 mediated prothrombin activation. Further, MASP-1 also failed to induce clotting by activation of prothrombin R320Q and S525A. Taken together, these results from the functional studies suggest that cleavage at R393 is a prerequisite for MASP-1 mediated clotting, nevertheless cleavage at R320 is needed for successful clotting. Since R320 is not processed by MASP-1 itself, any active thrombin species arising during prothrombin activation by MASP-1 must be responsible for cleavage at R320, supporting our model. This is coherent with the fact that the prothrombin active site mutant S525A is not able to induce clot formation when activated with MASP-1.

**Fig 2:**
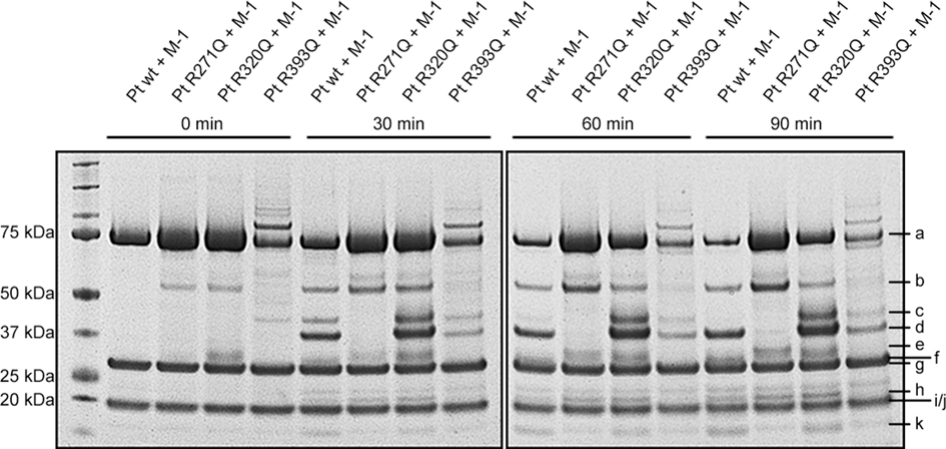
Time-course of the digestion of prothrombin wildtype and mutants by MASP-1. rMASP-1cf and the different prothrombin forms were incubated for up to 90 min. Bands were identified as a) uncleaved prothrombin, b) prethrombin-1, c) mIIR393 aa1-393, d) prethrombin-2, e) fragment F1.2 + 13 amino acids (up to R284), f) fragment F1.2, g) MASP-1 heavy chain, h) C-terminal part of thrombin heavy chain cleaved at R393, i) MASP-1 light chain, j) α-thrombinR393 light chain + heavy chain up to R393, k) fragment F1. Bands not indicated by letters are contaminations of the purification process or MASP-1 degradation products.

**Fig 3:**
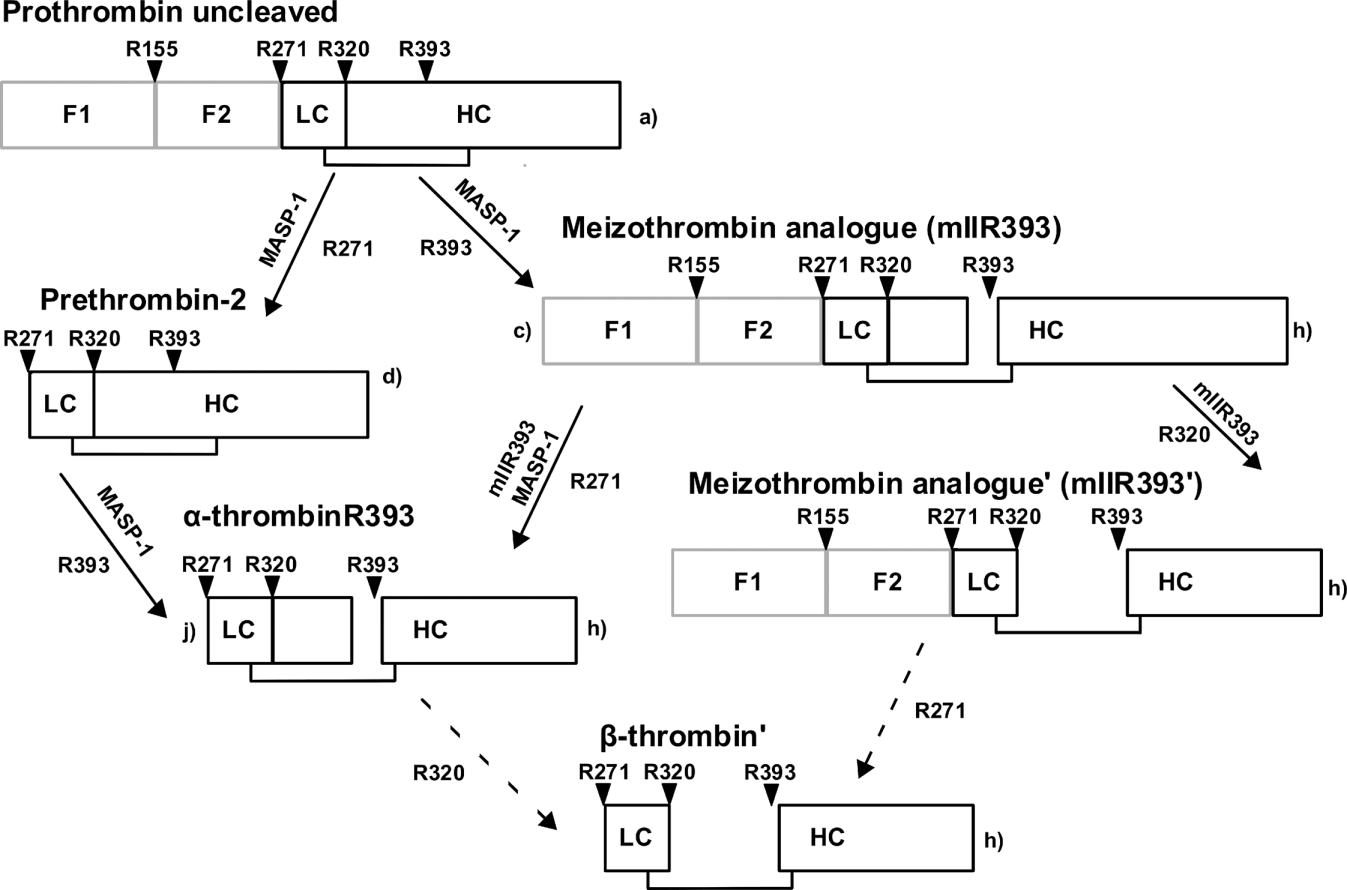
Proposed model of MASP-1 mediated prothrombin cleavage. There are two different pathways of prothrombin cleavage. MASP-1 cleaves first at R271, yielding prethrombin-2 which is subsequently cleaved at R393 to form the active species α-thrombinR393. When R393 is cleaved first, the active intermediate mIIR393 results. This intermediate is cleaved by MASP-1 (at R271) and itself (at R320) resulting in β-thrombin’ and mIIR393’, respectively. Further active species cannot be excluded. The small letters correspond to the nomenclature of the bands in Fig 2.

Our cleavage experiments reveal that MASP-1, similar to FXa, activates prothrombin via two possible pathways, however, MASP-1 gives rise to several novel thrombin species. Fig 2 shows the cleavage products of prothrombin and the prothrombin mutants digested by MASP-1 over a time course of up to 90 min. Fig 3 pictures our model of MASP-1 mediated prothrombin activation.

### 3.3. Cleavage pathway via prethrombin-2

In a first step, MASP-1 cleaves prothrombin at R271, yielding prethrombin-2 (Fig 2, band d) and prothrombin fragment F1.2 (Fig 2, band f). In a next step, prethrombin-2 is cleaved by MASP-1 at R393 which leads to the production of a novel species we termed α-thrombinR393 (Fig 2, bands h and j). Incubation of the prothrombin mutant R271Q with MASP-1 shows some cleavage at R284 (a secondary cleavage site of α-thrombin) instead of the blocked R271Q, yielding a slightly larger fragment F1.2 (Fig 2, band e) and a slightly smaller prethrombin-2. As MASP-1 shows a significantly lower cleavage rate at R284 in prothrombin than at R393 in prethrombin-2, there is no visible prethrombin-2 band in the R271Q digestion, instead this intermediate gets processed immediately by cleavage at R393 (Fig 2, band h; the corresponding truncated form of band j could not be detected).

### 3.4. Cleavage pathway via meizothrombin analogue (mIIR393)

The second pathway starts by cleavage of prothrombin at R393, yielding a novel species we called “meizothrombin analogue” (mIIR393) (Fig 2, bands c and h). In a next step, mIIR393 is processed at R271 which again yields α-thrombinR393 (Fig 2 bands h and j). This step can be performed by either MASP-1 or mIIR393 itself as we have shown earlier by inhibition of thrombin species with hirudin [8].

### 3.5. Further cleavage steps

The novel thrombin species (α-thrombinR393 and mIIR393) are putatively active and participate in further cleavage of prothrombin and intermediate cleavage products, therefore R271 and R393 no longer remain the only sites that are cleaved during MASP-1 mediated prothrombin activation. Recently, we suggested the existence of such active species based on experiments with hirudin which inhibits the activity of thrombin species but not MASP-1 [8]. Further evidence for the involvement of active thrombin species in this process now comes from the cleavage experiments with the R320Q prothrombin mutant. It can be observed that mIIR393 and prethrombin-2 (Fig 2, bands c and d) are accumulating. This suggests that, in contrast to MASP-1, α-thrombinR393 and/or mIIR393 have indeed cleavage activity towards R320, ultimately leading to the production of alternative forms mIIR393’ and β-thrombin’ and possibly even α-thrombin. However, the presence of those fragments could not be verified on the gel as they seem to be produced in small amounts and/or run along with fragments that are produced in significantly bigger amounts.

The cleavage at R155 (Fig 2, band b) which we had initially also identified as part of prothrombin activation by MASP-1, seems not to be a direct product of MASP-1 action. This cleavage product does not accumulate over time in presence of hirudin, thus it is more likely a product of prothrombin and mIIR393 auto degradation.

### 3.6. Involvement of active thrombin species

In FXa mediated prothrombin activation, several active thrombin species arise which themselves participate in the cleavage cascade. We therefore used the prothrombin active site mutant S525A to control for the contribution of active thrombin species in MASP-1 mediated prothrombin cleavage.

**Fig 4:**
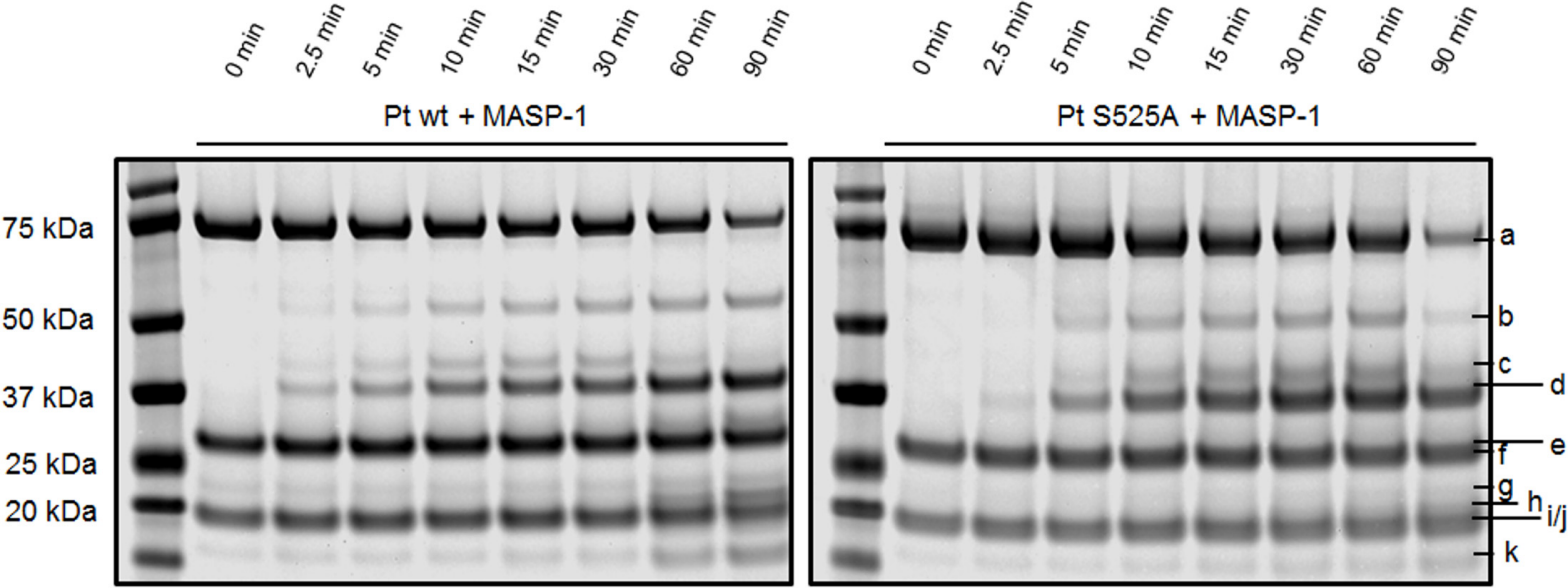
Time-course of the digestion of prothrombin wildtype and active site mutant S525A by MASP-1. rMASP-1cf and the different prothrombin forms were incubated for up to 90 min. Bands were identified as a) uncleaved prothrombin, b) prethrombin-1, c) mIIR393 aa1-393, d) prethrombin-2, e) fragment F1.2, f) MASP-1 heavy chain, g) degradation product of the MASP-1 heavy chain, h) C-terminal part of thrombin heavy chain cleaved at R393, i) MASP-1 light chain, j) α-thrombinR393 light chain + heavy chain up to R393, k) fragment F1. Bands not indicated by letters are contaminations of the purification process.

Fig 4 shows the different cleavage pattern we obtained by comparing incubation of MASP-1 with wildtype prothrombin and the mutant. The cleavage pattern of MASP-1 incubated with S525A shows a slower degradation of mIIR393 (Fig 4, band c) which is likely due to the fact, that this intermediate is mostly processed by itself at R271 and R320, whereas MASP-1 cleaves at R271 only. Also the intermediate F1.2 (Fig 4, band e) is much less prominent. The reason for the smaller amount of F1.2 in the S525A mutant compared with wildtype prothrombin may be that in the wildtype form the active species mIIR393, and not MASP-1, is mainly responsible for cleavage at R271. Additionally, mIIR393 may also exhibit a certain amount of back-cleavage on full-length prothrombin. Further it can be observed that the C-terminal part of the thrombin heavy chain cleaved at R393 (Fig 4, band h) is less prominent. This suggests that some active thrombin species cleave at R393 as well.

**Fig 5:**
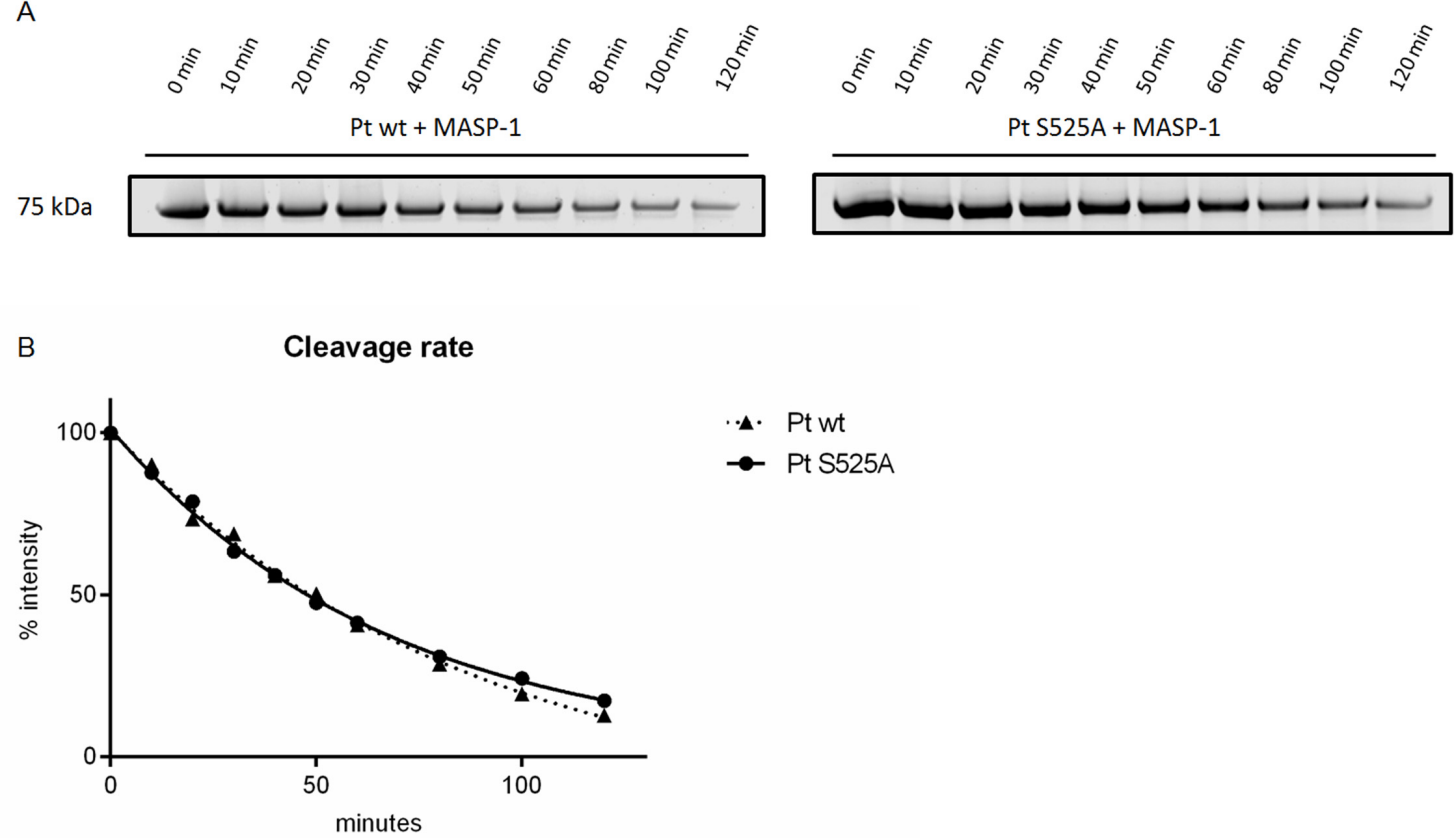
Kinetic analysis of prothrombin cleavage by MASP-1. A, SDS-PAGE of prothrombin wildtype or the mutant form S525A (both 1.4μM) digested by MASP-1 (1.76 μM) over 120 min. B, curves fitted by non-linear regression.

The decrease of the prothrombin band (Fig 4, band a) did not seem to differ between prothrombin wildtype and the active site mutant. To further evaluate this, we have calculated pseudo first order rate constants to compare the kinetics of MASP-1 mediated decrease of wildtype prothrombin and the prothrombin active site mutant S525A (Fig 5).

The k_obs_/[E] (observed k) values for both prothrombin forms cleaved by MASP-1 were not significantly different (141.0*10^3^M^-1^s^-1^ for the wildtype form and 136.9*10^3^M^-1^s^-1^ for the mutant). However, the fitted curves (Fig 5B) show that after 80 min the active site mutant prothrombin is processed more slowly than the wildtype form. This indicates that there is autocatalytical cleavage of thrombin species involved in MASP-1 mediated prothrombin activation, nevertheless it seems to occur to a rather small extent and only in the late phase of the process when thrombin species become more abundant.

Taken together, all these results support the important role of active thrombin species in MASP-1 induces prothrombin activation.

## Discussion

In the last decade the interplay between the complement system and blood coagulation has raised increasing interest and a growing number of interactions have been discovered [18]. MASP-1 in particular has numerous interactions with the coagulation system [4]. Nevertheless, many of the underlying mechanisms of how MASP-1 influences the coagulation system and the relevance of these interactions are not yet completely understood. In the present study we show for the first time a model of MASP-1 mediated prothrombin activation which was developed by using prothrombin mutants R271Q, R320Q, and R393Q.

At first glance, prothrombin cleavage by MASP-1 strongly resembles FXa mediated prothrombin activation. Similar to FXa mediated prothrombin cleavage, there are two different pathways, both ending up in the production of a common thrombin species [19]. Both enzymes are able to cleave at position R271, however, they have different preferences regarding the specific cleavage site that activates prothrombin between the heavy and the light chain: FXa cleaves at R320 whereas MASP-1 cleaves 73 amino acids further upstream at R393. Nevertheless, both cleavage sites are located within the interchain disulfide-bridge (C292-C438), therefore cleavage at R393 does not lead to dissociation of the heavy and light chains.

In FXa mediated prothrombin cleavage, preference for the first cleavage site depends on its environment: In the presence of an assembling prothrombinase complex (requires a membrane surface), R320 becomes the preferred cleavage site, which leads to the production of meizothrombin, an intermediate that acts anticoagulant. On the other hand, when FXa activates prothrombin in absence of a prothrombinase complex (typical for fluid phase activation), R271 becomes the preferred site for the first cleavage, yielding prethrombin-2 [20,21]. In contrast, MASP-1 does not exhibit a clear preference for a specific first cleavage site in experiments performed in solution; R271 and R393 are selected at similar rates.

Meizothrombin (mIIa) and its MASP-1 cleaved analogue (mIIR393) are both active species. It is known that mIIa has an impaired ability to clot fibrinogen and even has anticoagulant effects by binding thrombomodulin and activating protein C [22]. Its analogue mIIR393 is likely to exhibit an impaired clotting ability as well. Evidence comes from the thrombelastographic experiments with the prothrombin mutant R320Q. As mIIR393 accumulates, there would be plenty of this intermediate available for clot formation, but clot formation does not occur. This finding also implies that mIIR393 is able to cleave itself at R320 which then leads to production of the intermediate mIIR393’.

The finding that the cleavage site R320 is necessary for MASP-1 induced clotting suggests that the other novel thrombin species, α-thrombinR393, is not sufficiently capable to induce fibrin clotting either. Since prethrombin-2 also accumulates when R320 is abolished, it is possible that either α-thrombinR393 or mIIR393 cleave prethrombin-2 at R320 producing α-thrombin. However, since the α-thrombin heavy chain would run along with the vast amount of the MASP-1 heavy chain on the gel, it was not possible to isolate this band.

Our thrombelastographic experiments confirm that cleavage at R393 is required but not sufficient for MASP-1 mediated clotting, since not only blockage of R393 but also blockage of R320 prevents clot formation. As R393 is cleaved earlier as R320, this suggests that the action of mIIR393 or α-thrombinR393 is necessary for cleavage at R320 to yield the active species ultimately responsible for fibrin clotting. This is further supported by the absence of clot formation when the active site mutant S525A was used.The cleavage assay with the active site mutant S525A showed that the arising thrombin species are involved and have at least partly the same cleavage sites as MASP-1. The most prominent difference in the active site mutant is the accumulation of mIIR393 (Fig 4, band c), which also seems to be a crucial player in the MASP-1 mediated prothrombin activation. mIIR393 does not only exert cleavage action on itself, it most likely cleaves mIIR393’ and α-thrombinR393 as well. Since the C-terminal part of the thrombin heavy chain, cleaved at R393, is produced less when the mutant S525A is used, it can be assumed that at least some thrombin species cleave at R393 as well.

It is further notable that the degradation product of the MASP-1 heavy chain (Fig 4, band g) is produced to a smaller extent during the digestion of the active site mutant prothrombin. This indicates that MASP-1 is a target for some active thrombin species. Assuming that thrombin-degraded MASP-1 is inactive, this could explain why the cleavage rates of prothrombin wildtype and active site mutant do not differ significantly: while autocatalytic cleavage by active thrombin species does not occur in the active site mutant, there is at the same time more active MASP-1 available.

It is also important to mention that the production of F1 (Fig 4, band k) is impaired in the digestion of the active site mutant prothrombin. This confirms that R155 is mainly cleaved by thrombin species but is not a good cleavage site for MASP-1.

Prothrombin contains two electropositive exosites termed anion binding exosite I and anion binding exosite II (ABEI and ABEII). Both exosites are important players in the recognition and the binding of specific substrates, effectors and inhibitors. ABEI is located next to the active site cleft and is involved in the recognition and binding of fibrinogen, hirudin and FV [23, 24, 25]. The majority of the ABEI residues are located on the heavy chain of prothrombin. Cleavage at R155, R271, R320 or R393 individually does not disrupt this exosite. However, cleavage at both R320 and R393 (yielding β-thrombin, β-thrombin’ or mIIR393’) leads to a loss of five ABEI residues which are located between R382 and R393, the fragment lost in β-thrombin production. The loss of this fragment results in a remarkable decrease of the efficiency of thrombin species to process fibrinogen and therefore a reduced clotting activity [13]. This implies that MASP-1 mediated clot formation is mainly driven by α-thrombinR393 and mIIR393, because β-thrombin’ and mIIR393’ lack a functional exosite I.

FXa binds to membranes in a calcium dependent matter to form the prothrombinase complex (in combination with FVa) which enhances the prothrombin activation significantly [26]. Since MASP-1 also contains a Ca^2+^ dependent binding domain (EGF), it is possible that MASP-1 mediated prothrombin activation could be enhanced in the presence of phospholipids and Ca^2+^. However, because our recombinant form of MASP-1 lacks the CUB-EGF-CUB domains, it is not possible to test for an enhancing effect of phospholipids with our enzyme.

In summary, we have shown for the first time that MASP-1 activates prothrombin via two simultaneous pathways which seem equally preferred in the fluid phase. Furthermore, we have shown the existence of novel alternative thrombin forms we called α-thrombinR393, meizothrombin analogue (mIIR393), and β-thrombin’ which must be active in the process of MASP-1 mediated prothrombin cleavage. Even though the cleavage site R393 is necessary to induce clot formation, it is not sufficient by itself but it needs the support of the cleavage site R320 which is the activation site of α-thrombin. It is important to mention that the experiments were performed in purified systems that lack the effects of inhibitory and enhancing components that can be found in whole blood. However, we have recently shown that MASP-1 does show significant effects on clot formation in whole blood and plasma [8].

Our results do not suggest that MASP-1 competes with FXa for prothrombin activation. FXa as part of the prothrombinase complex is the activator of prothrombin in the coagulation cascade and it is of course much stronger and more efficient in activating prothrombin compared with MASP-1. However, we do believe that under certain pathophysiological conditions activated MASP-1 supports or even triggers clot formation and is able to sustain it beyond the normal coagulation activation pathways. An example may be diabetes, both type 1 and type 2 are recognised to have a strong inflammatory component. We have recently shown that MASP-1 plasma levels are elevated in patients with type 1 diabetes [27]. In this inflammatory environment, more MASP-1 may activate and lead to both activation of the complement lectin pathway as well as to low level prothrombin activation and fibrin formation. In this way MASP-1 could contribute to thrombotic complications which are frequent in diabetes and other inflammatory diseases.

## Abbreviations

MBL: mannan-binding lectin
MASP: mannan-binding lectin-associated serine protease
FXIII: factor XIII
FX: factor X
rMASP-1cf: MASP-1 catalytic fragment
MES: 2-(N-Morpholino)- ethanesulfonic acid
PVDF: polyvinylidene difluoride
FXa: activated factor X
CT: clotting time
k_obs_: k observed
F1.2: prothrombin fragment 1+2
mIIa: meizothrombin
mIIR393: meizothrombin analogue cleaved at R393
ABEI/ABEII: anion binding exosite I/II
